# Supplementary material for: Analysis of drought and heat stress response genes in rice using co-expression network and differentially expressed gene analyses
Source: PeerJ. 2024 Apr 30;12:e17255. doi: 10.7717/peerj.17255 (PMC11067907; doi:10.7717/peerj.17255)
Supplement: Supplemental Information 1 [file peerj-12-17255-s001.docx]

MIQE checklist

| **Item to check** | **Importance** | **Item to check** | **Importance** |
| --- | --- | --- | --- |
| *1.Experimental design* |  | *6.qPCR oligonucleotides* |  |
| 1.1 Definition of experimental and control groups | √ | 6.1 Primer sequences | √ |
| 1.2 Number within each group | √ | RTPrimerDB identification number | - |
| Assay carried out by the core or investigator’s laboratory? | - | Probe sequences | - |
| Acknowledgment of authors’ contributions | - | 6.2 Location and identity of any modifications | √ |
| *2.Sample* |  | Manufacturer of oligonucleotides | - |
| 2.1 Description | √ | Purification method | - |
| 2.2 Volume/mass of sample processed | √ | *7.qPCR protocol* |  |
| 2.3 Microdissection or macrodissection | √ | 7.1 Complete reaction conditions | √ |
| 2.4 Processing procedure | √ | 7.2 Reaction volume and amount of cDNA/DNA | √ |
| 2.5 If frozen, how and how quickly? | √ | 7.3 Primer, (probe), Mg2, and dNTP concentrations | √ |
| 2.6 If fixed, with what and how quickly? | √ | 7.4 Polymerase identity and concentration | √ |
| 2.7 Sample storage conditions and duration (especially for FFPEb samples) | √ | 7.5 Buffer/kit identity and manufacturer | √ |
| *3.Nucleic acid extraction* |  | Exact chemical composition of the buffer | - |
| 3.1 Procedure and/or instrumentation | √ | 7.6 Additives (SYBR Green I, DMSO, and so forth) | √ |
| 3.2 Name of kit and details of any modifications | √ | Manufacturer of plates/tubes and catalog number | - |
| Source of additional reagents used | - | 7.7 Complete thermocycling parameters | √ |
| 3.3 Details of DNase or RNase treatment | √ | Reaction setup (manual/robotic) | - |
| 3.4 Contamination assessment (DNA or RNA) | √ | 7.8 Manufacturer of qPCR instrument | √ |
| 3.5 Nucleic acid quantification | √ | *8.qPCR validation* |  |
| 3.6 Instrument and method | √ | Evidence of optimization (from gradients) | - |
| Purity (A260/A280) | - | 8.1 Specificity (gel, sequence, melt, or digest) | √ |
| Yield | - | 8.2 For SYBR Green I, Cq of the NTC | √ |
| 3.7 RNA integrity: method/instrument | √ | 8.3 Calibration curves with slope and y intercept | √ |
| 3.8 RIN/RQI or Cq of 3 and 5 transcripts | √ | 8.4 PCR efficiency calculated from slope | √ |
| Electrophoresis traces | - | CIs for PCR efficiency or SE | - |
| 3.9 Inhibition testing (Cq dilutions, spike, or other) | √ | 8.5 r2 of calibration curve | √ |
| *4.Reverse transcription* |  | 8.6 Linear dynamic range | √ |
| 4.1 Complete reaction conditions | √ | 8.7 Cq variation at LOD | √ |
| 4.2 Amount of RNA and reaction volume | √ | CIs throughout range | - |
| 4.3 Priming oligonucleotide (if using GSP) and concentration | √ | 8.8 Evidence for LOD | √ |
| 4.4 Reverse transcriptase and concentration | √ | 8.9 If multiplex, efficiency and LOD of each assay | √ |
| 4.5 Temperature and time | √ | *9.Data analysis* |  |
| Manufacturer of reagents and catalogue numbers | - | 9.1 qPCR analysis program (source, version) | √ |
| Cqs with and without reverse transcription | - | 9.2 Method of Cq determination | √ |
| Storage conditions of cDNA | - | 9.3 Outlier identification and disposition | √ |
| *5.qPCR target information* |  | 9.4 Results for NTCs | √ |
| 5.1 Gene symbol | √ | 9.5 Justification of number and choice of reference genes | √ |
| 5.1 Sequence accession number | √ | 9.6 Description of normalization method | √ |
| Location of amplicon | - | Number and concordance of biological replicates | - |
| 5.1 Amplicon length | √ | 9.7 Number and stage (reverse transcription or qPCR) of technical replicates | √ |
| 5.1 In silico specificity screen (BLAST, and so on) | √ | 9.8 Repeatability (intraassay variation) | √ |
| Pseudogenes, retropseudogenes, or other homologs? | - | Reproducibility (interassay variation, CV) | - |
| Sequence alignment | - | Power analysis | - |
| Secondary structure analysis of amplicon | - | 9.9 Statistical methods for results significance | √ |
| 5.2 Location of each primer by exon or intron (if applicable) | √ | 9.10 Software (source, version) | √ |
| 5.3 What splice variants are targeted? | √ | Cq or raw data submission with RDML | - |

# 1. Experimental design

## 1.1 Definition of experimental and control groups:

| Primer location | 1 | 2 | 3 | 4 | 5 | 6 | 7 | 8 | 9 |
| --- | --- | --- | --- | --- | --- | --- | --- | --- | --- |
| A | eEF1 | BAG6 | HSP23.2 | OsDjC53 | MBF1C | HSP21.9 | UGT83A1 | OsCPn60a1 | eEF1 |
| B | eEF1 | BAG6 | HSP23.2 | OsDjC53 | MBF1C | HSP21.9 | UGT83A1 | OsCPn60a1 | eEF1 |
| C | eEF1 | BAG6 | HSP23.2 | OsDjC53 | MBF1C | HSP21.9 | UGT83A1 | OsCPn60a1 | eEF1 |
| D | eEF1 | BAG6 | HSP23.2 | OsDjC53 | MBF1C | HSP21.9 | UGT83A1 | OsCPn60a1 | eEF1 |
| E | eEF1 | BAG6 | HSP23.2 | OsDjC53 | MBF1C | HSP21.9 | UGT83A1 | OsCPn60a1 | |
| F | eEF1 | BAG6 | HSP23.2 | OsDjC53 | MBF1C | HSP21.9 | UGT83A1 | OsCPn60a1 | |
| G | eEF1 | BAG6 | HSP23.2 | OsDjC53 | MBF1C | HSP21.9 | UGT83A1 | OsCPn60a1 | |
| H | eEF1 | BAG6 | HSP23.2 | OsDjC53 | MBF1C | HSP21.9 | UGT83A1 | OsCPn60a1 | |

| Sample location | 1 | 2 | 3 | 4 | 5 | 6 | 7 | 8 | 9 |
| --- | --- | --- | --- | --- | --- | --- | --- | --- | --- |
| A | Control1 | Control1 | Control1 | Control1 | Control1 | Control1 | Control1 | Control1 | Control1 |
| B | Control2 | Control2 | Control2 | Control2 | Control2 | Control2 | Control2 | Control2 | Control2 |
| C | Control3 | Control3 | Control3 | Control3 | Control3 | Control3 | Control3 | Control3 | Control3 |
| D | Control4 | Control4 | Control4 | Control4 | Control4 | Control4 | Control4 | Control4 | Control4 |
| E | Heat1 | Heat1 | Heat1 | Heat1 | Heat1 | Heat1 | Drought1 | Drought1 |  |
| F | Heat2 | Heat2 | Heat2 | Heat2 | Heat2 | Heat2 | Drought2 | Drought2 |  |
| G | Heat3 | Heat3 | Heat3 | Heat3 | Heat3 | Heat3 | Drought3 | Drought3 |  |
| H | Heat4 | Heat4 | Heat4 | Heat4 | Heat4 | Heat4 | Drought4 | Drought4 |  |

## 1.2 Number within each group

Four biological replicates were set for each stress type or control, and the tissue materials were derived from different plant individuals. The testing is done independently by our laboratory.

# 2. Sample

## 2.1 Description

Oryza Sativa, Jiahe 102 (State Key Laboratory of Hybrid Rice, Wuhan, China) was used as a model rice variety subjected to appropriate environmental conditions, drought stress, and heat stimulation and for RNA extraction for quantitative real-time PCR (qRT-PCR).

The seed coat was removed, sterilized, and soaked in a dark room at 37 °C for 24 h. The seeds were allowed to germinate at 25 °C in the dark for 48 h and then transferred to a culture bottle for cultivation in the Murashige and Skoog (MS) medium at 25 °C for 14 days. The control group was directly sampled. For the drought treatment group, on the evening of the 13th day, the samples were cultured for 12 h in the MS medium containing 0.3 M mannitol. For the heat treatment group, on the 14th day, rice seedlings cultured in normal MS medium were exposed to a temperature of 37 °C for 1 h and then sampled.

## 2.2 Volume/mass of sample processed

More than 4 tubes of whole shoot tissues were collected per material for each treatment (including control). Take 100 mg per tube of material.

## 2.3 Microdissection or microdissection

Take the Whole shoot tissues with sterilized scissors, cut slightly and weigh.

## 2.4 Processing procedure

Whole shoot tissues (100 mg) of rice from different treatment groups were weighed and placed in a grinding tube containing steel beads. The next step of freezing should be promptly executed.

## 2.5 If frozen, how and how quickly?

The grinding tubes were immersed in liquid nitrogen for 10–20 min.

## 2.6 If fixed, with what and how quickly?

No fixed.

## 2.7 Sample storage conditions and duration (especially for FFPEb samples)

The samples are stored in either liquid nitrogen or at a temperature of -80 ℃.

# 3. Nucleic acid extraction

## 3.1 Procedure and/or instrumentation

Tissuelyser® is used for grinding (Jingxin, Shanghai, China). RNA extraction was performed using the FastPure Universal Plant Total RNA Isolation Kit (Vazyme, Nanjing, China), and the extracted total RNA was stored at −80 °C.

## 3.2 Name of kit and details of any modifications

FastPure Universal Plant Total RNA Isolation Kit (Vazyme, Nanjing, China).

## 3.3 Details of DNase or RNase treatment

The equipment or tubes used in the experiment were pretreated by means of double sterilization or DEPC water immersion to reduce the contamination of RNase.

## 3.4 Contamination assessment (DNA or RNA)

OD values of 230nm, 260nm and 280nm were used to detect sample quality. The A260/A280 of the RNA samples used in this experiment were all between 1.9 and 2.1. The A260/A230 ratio is greater than 2.0.

## 3.5 Nucleic acid quantification

The OD values of 230nm, 260nm and 280nm were used for nucleic acid quantification. The RNA concentration of the samples used in this experiment was between 100-200ng/μl.

## 3.6 Instrument and method

Use NANO-200 ultramicro nucleic acid analyzer (AoSheng, Hangzhou, China) for OD values detecting.

## 3.7 RNA integrity: method/instrument

OD values of 230nm, 260nm and 280nm were used to detect sample quality. The A260/A280 of the RNA samples used in this experiment were all between 1.9 and 2.1. The A260/A230 ratio is greater than 2.0.

## 3.8 RIN/RQI or Cq of 3‘ and 5‘ transcripts

This test is not performed due to limited conditions.

## 3.9 Inhibition testing (Cq dilutions, spike, or other)

This test is not performed due to limited conditions

# 4. Reverse transcription

## Complete reaction conditions

cDNA was synthesized using the Revert Aid First Strand cDNA Synthesis Kit (Thermo Scientific, Watham, MA, USA).

4.1.1 Add the following reagents into a sterile, nucleasefree tube on ice in the indicated order:

| Total RNA | 0.1 ng - 5 µg |
| --- | --- |
| Oligo (dT)_18_ primer | 1 µL |
| Water, nuclease-free | to 12 µL |

4.1.2 ***Optional****.* If the RNA template is GC-rich or contains secondary structures, mix gently, centrifuge briefly and incubate at 65°C for 5 min. Chill on ice, spin down and place the vial back on ice.

4.1.3 Add the following components in the indicated order: Mix gently and centrifuge briefly.

| 5X Reaction Buffer | 4 µL |
| --- | --- |
| RiboLock RNase Inhibitor (20 U/µL) | 1 µL |
| 10 mM dNTP Mix | 2 µL |
| RevertAid M-MuLV RT (200 U/µL) | 1 µL |
| Total volume | 20 µL |

4.1.4 For oligo(dT)_18_ or gene-specific primed cDNA synthesis, incubate for 60 min at 42°C.

4.1.5 For random hexamer primed synthesis, incubate for 5 min at 25°C followed by 60 min at 42°C. **Note.** For GC-rich RNA templates the reaction temperature can be increased up to 45°C.

4.1.6 Terminate the reaction by heating at 70°C for 5 min.

4.1.7 The reverse transcription reaction product can be directly used in PCR applications or stored at -20°C for less than one week. For longer storage, -70°C is recommended.

## 4.2 Amount of RNA and reaction volume

0.1 ng - 5 µg/20 µL.

## 4.3 Priming oligonucleotide (if using GSP) and concentration

Oligo (dT)_18_ primer, 1 µL/20 µL.

## 4.4 Reverse transcriptase and concentration

RevertAid M-MuLV RT (Thermo Scientific, Watham, MA, USA), 200U/20 µL

## 4.5 Temperature and time

Incubate for 60 min at 42°C. Terminate the reaction by heating at 70°C for 5 min.

# 5. qPCR target information

## 5.1 Gene symbol, Sequence accession number, Amplicon length, and In silico specificity screen (BLAST, and so on)

| Gene symbol | Sequence accession number | Amplicon length | BLAST |
| --- | --- | --- | --- |
| eEF1 | LOC_Os03g08010 | 127 | Amplification sequence unique |
| BAG6 | LOC_Os02g15930 | 146 | Amplification sequence unique |
| HSP23.2 | LOC_Os04g36750 | 124 | Amplification sequence unique |
| OsDjC53 | LOC_Os06g09560 | 80 | Amplification sequence unique |
| MBF1C | LOC_Os06g39240 | 107 | Amplification sequence unique |
| HSP21.9 | LOC_Os11g13980 | 139 | Amplification sequence unique |
| UGT83A1 | LOC_Os03g55030 | 143 | Amplification sequence unique |
| OsCPn60a1 | LOC_Os12g17910 | 103 | Amplification sequence unique |

## 5.2 Location of each primer by exon or intron (if applicable)

All 5 'primers and 3' primers span exons.

## 5.3 What splice variants are targeted?

None.

# 6. qPCR oligonucleotides

## 6.1 Primer sequences

| Gene name | MSU-ID | Group | 5' primer | 3' primer |
| --- | --- | --- | --- | --- |
| eEF1 | LOC_Os03g08010 | Reference | GATGATCTGCTGCTGCAACAAG | GGGAATCTTGTCAGGGTTGTAG |
| BAG6 | LOC_Os02g15930 | Green | GTTGAAAGTAGTGTGTCAGCT | AAGGATACTGATGAGTCCCC |
| HSP23.2 | LOC_Os04g36750 | Green | GGTGGAGGTGGAGGACAA | CCAGAACCTGCCGTAGGA |
| OsDjC53 | LOC_Os06g09560 | Green | GATTTCCTCGGCGAGATGG | ACGAACAGCTGCTGCAA |
| MBF1C | LOC_Os06g39240 | Green | AGGTTGAGCGGCAACATC | CGCATCGCCTGGTTCAC |
| HSP21.9 | LOC_Os11g13980 | Green | CGTACGGCTACGGCTACAT | TCCTTCCAGTCGCACCTC |
| UGT83A1 | LOC_Os03g55030 | Darkmagenta | GGCGTCCTCAACGAGAAG | CAGACGAGGTCGAAGATGATG |
| OsCPn60a1 | LOC_Os12g17910 | Darkmagenta | CAAGGCTGTCCTTCAGGATATT | TGTCCCAAGTTGCTCTTCAG |

## 6.2 Location and identity of any modifications

None.

# 7. qPCR protocol

## 7.1 Complete reaction conditions

The reaction system was configured with SYBR™ Green PCR Master Mix (Thermo Scientific, Watham, MA, USA, [Document Connect (thermofisher.cn)](https://www.thermofisher.cn/document-connect/document-connect.html?url=https://assets.thermofisher.cn/TFS-Assets%2FLSG%2Fmanuals%2Fcms_041053.pdf)) and all steps were performed according to the instructions. The reaction system is formulated as follows:

| **component** | **Volume (µL)** | **Final Concentration** |
| --- | --- | --- |
| 2X SYBR^®^ Green PCR Master Mix | 5 | 1X |
| Forward Primer | 0.25 | 50 nM |
| Reverse Primer | 0.25 | 50 nM |
| Template | 0.5 | 1ng to 100 ng |
| Water | 4.5 | — |
| Total | 10 | — |

qRT-PCR analysis was performed using a LightCycler 96 (Roche, Basel, Switzerland).

| **Step** | **Polymerase Activation** | **PCR** | | |
| --- | --- | --- | --- | --- |
|  | HOLD | CYCLE (40 cycles) | | |
|  |  | Denature | Anneal/Extend |  |
| Temp. | 95.0 °C | 95.0 °C | 60.0 °C |  |
| Time | 10 min | 15 sec | 1 min |  |
| Volume |  | 10 µL | | |

## 7.2 Reaction volume and amount of cDNA/DNA

Reaction volume is 10 µL, and amount of cDNA is 0.5 µL (1ng to 100 ng) per test.

## 7.3 Primer, (probe), Mg^2+^, and dNTP concentrations

The final primer concentration was 50 nM, using the preset Mg2+ and dNTP concentrations due to the use of SYBR™ Green PCR Master Mix.

## 7.4 Polymerase identity and concentration

See SYBR™ Green PCR Master Mix (Thermo Scientific, Watham, MA, USA) for details. Specification.

## 7.5 Buffer/kit identity and manufacturer

SYBR™ Green PCR Master Mix (Thermo Scientific, Watham, MA, USA).

## 7.6 Additives (SYBR Green I, DMSO, and so forth)

SYBR Green I.

## 7.7 Complete thermocycling parameters

| **Step** | **Polymerase Activation** | **PCR** | | |
| --- | --- | --- | --- | --- |
|  | HOLD | CYCLE (40 cycles) | | |
|  |  | Denature | Anneal/Extend |  |
| Temp. | 95.0 °C | 95.0 °C | 60.0 °C |  |
| Time | 10 min | 15 sec | 1 min |  |
| Volume |  | 10 µL | | |

## 7.8 Manufacturer of qPCR instrument

qRT-PCR analysis was performed using a LightCycler 96 (Roche, Basel, Switzerland).

# 8. qPCR validation

## 8.1 Specificity (gel, sequence, melt, or digest)

The specificity was reflected by the melt curve. (See Supplementary2, “RAW.lc96p”)

## 8.2 For SYBR Green I, Cq of the NTC

NTC were not used, because relative quantification was performed using internal reference genes.

## 8.3 Calibration curves with slope and y intercept

Standard curves were not used, beacuse relative quantification was performed using internal reference genes.

## 8.4 PCR efficiency calculated from slope

Standard curves were not used, beacuse relative quantification was performed using internal reference genes.

## 8.5 r2 of calibration curve

Standard curves were not used, beacuse relative quantification was performed using internal reference genes.

## 8.6 Linear dynamic range

Standard curves were not used, beacuse relative quantification was performed using internal reference genes.

## 8.7 Cq variation at LOD

The calculation is carried out by the software of the instrument.

## 8.8 Evidence for LOD

The calculation is carried out by the software of the instrument.

## 8.9 If multiplex, efficiency and LOD of each assay

The calculation is carried out by the software of the instrument.

# 9. Data analysis

## 9.1 qPCR analysis program (source, version)

LightCycler® QC Test Software 3.0 (Roche, Basel, Switzerland). [LightCycler (roche.com)](https://lifescience.roche.com/global/en/products/product-category/lightcycler.html#4)

## 9.2 Method of Cq determination

Use the software default method.

## 9.3 Outlier identification and disposition

Use the software default method.

## 9.4 Results for NTCs

NTC were not used, because relative quantification was performed using internal reference genes.

## 9.5 Justification of number and choice of reference genes

*eEF1* was used as the reference gene, because this is an internal reference gene that is commonly used in rice.

## 9.6 Description of normalization method

Use the software default method.

## 9.7 Number and stage (reverse transcription or qPCR) of technical replicates

Each sample is independent from RNA extraction to qPCR detection.

## 9.8 Repeatability (intraassay variation)

Due to the small size of the plant and the limited amount of tissue, each sample was from 3 different individuals. More than 4 samples were extracted from each treatment (including controls) and were biologically replicated for each other in subsequent experiments.

## 9.9 Statistical methods for results significance

The method of T test was used to detect the significance of this experiment.

## 9.10 Software (source, version)

LightCycler® QC Test Software 3.0 (Roche, Basel, Switzerland). [LightCycler (roche.com)](https://lifescience.roche.com/global/en/products/product-category/lightcycler.html#4); Graphpad Prism 9.5.1; Microsoft® Excel® For Microsoft 365MSO (2310 Build 16.0.16924.20002).
