# Supplementary figures and images for: Analysis of drought and heat stress response genes in rice using co-expression network and differentially expressed gene analyses
Source: PeerJ. 2024 Apr 30;12:e17255. doi: 10.7717/peerj.17255 (PMC11067907; doi:10.7717/peerj.17255)

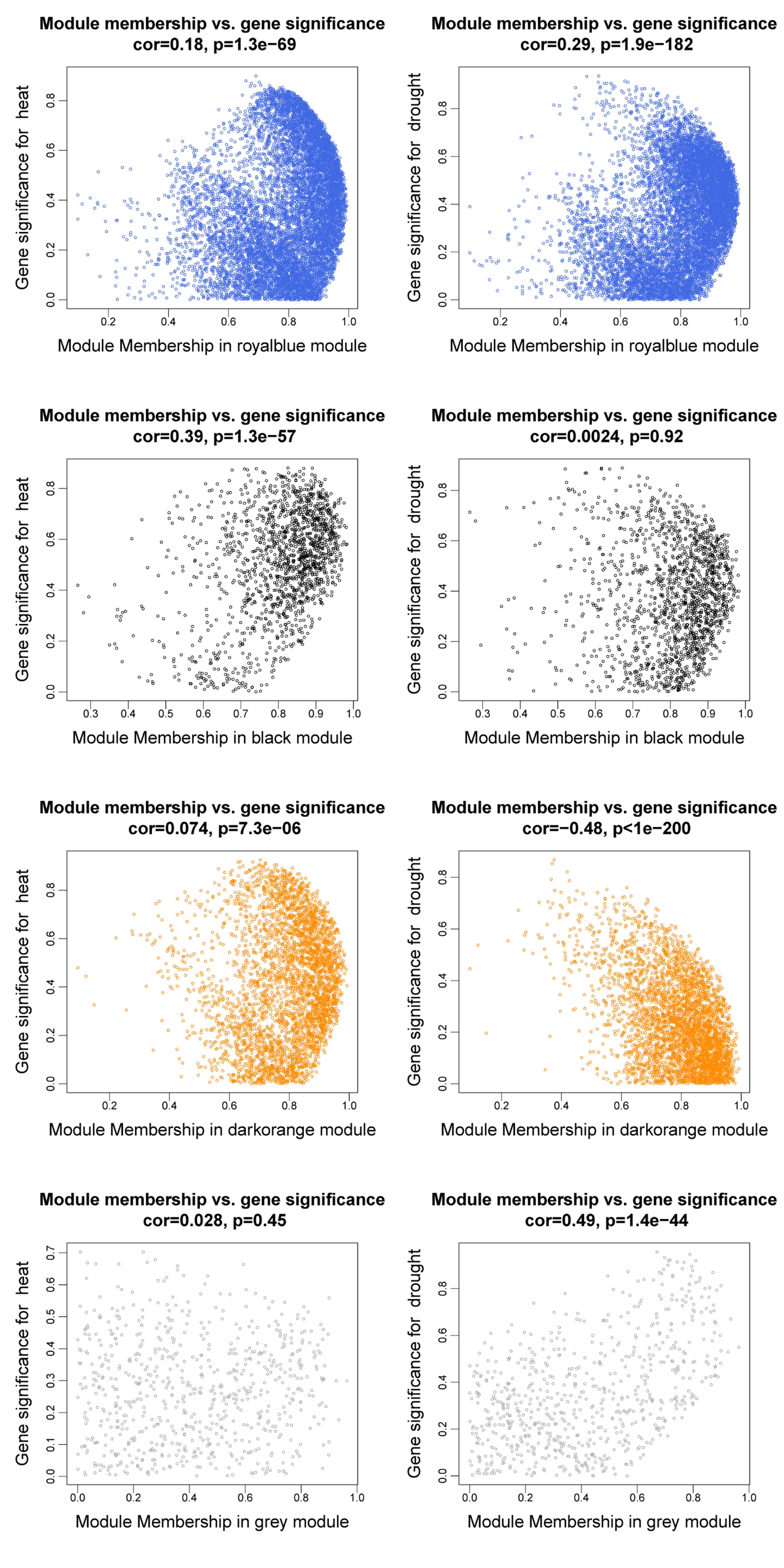

Supplement: Supplemental Information 4 — Each gene is indicated by a hollow dot. The x-axis represents the correlation between the module eigengene and the gene expression profile in the different color groups. The y-axis shows the correlation between the gene and different degrees of heat or drought stress. [file peerj-12-17255-s004.png]

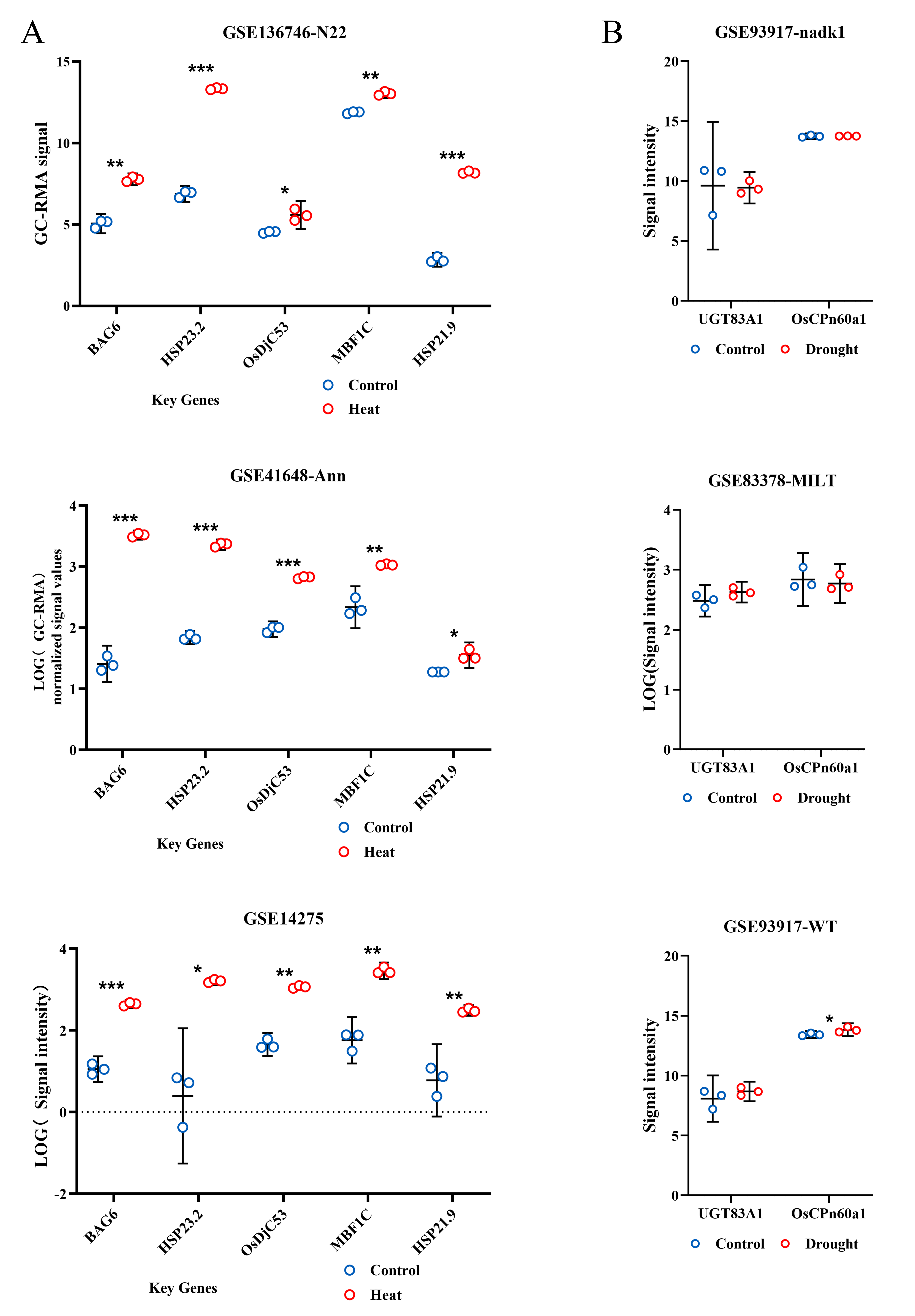

Supplement: Supplemental Information 5 — Gene names are plotted on the x-axis, and gene expression levels are shown on the y-axis. (A) The top, middle, and bottom figures show the three datasets related to heat stress. (B) Drought stress. [file peerj-12-17255-s005.png]

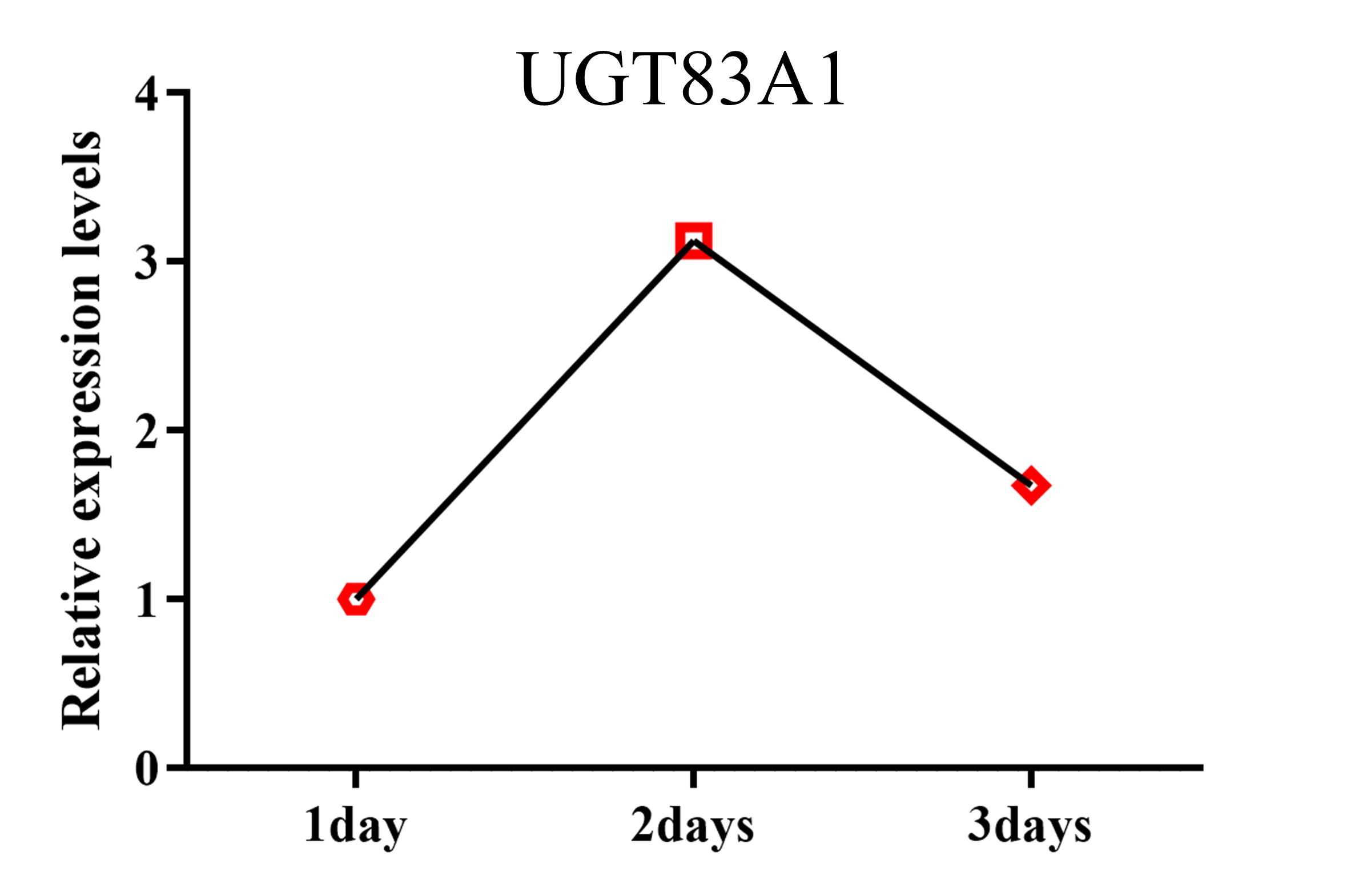

Supplement: Supplemental Information 6 — The x-axis represents the duration of drought stress treatment, and the y-axis represents the relative expression levels of genes. [file peerj-12-17255-s006.png]

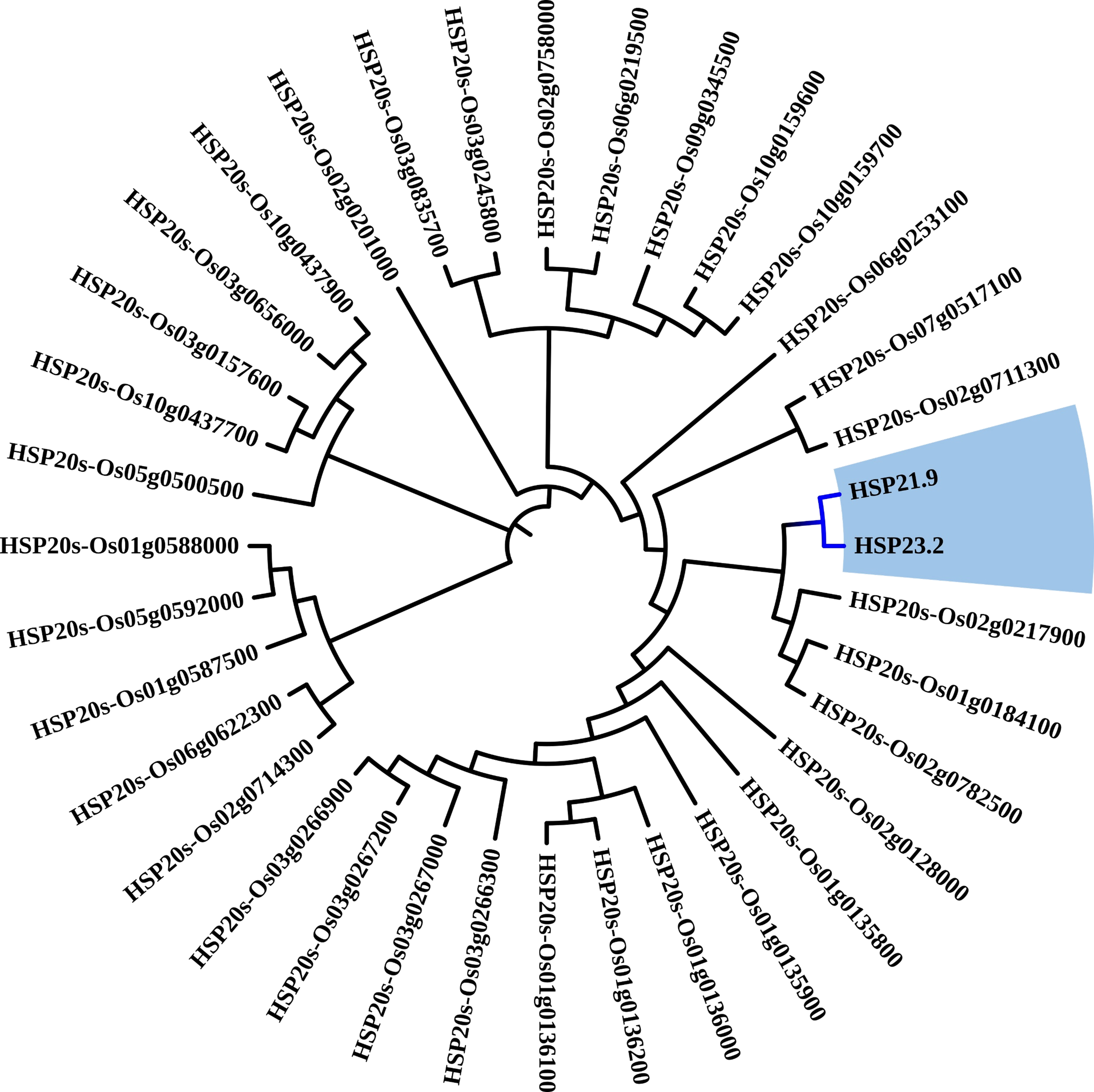

Supplement: Supplemental Information 7 [file peerj-12-17255-s007.png]

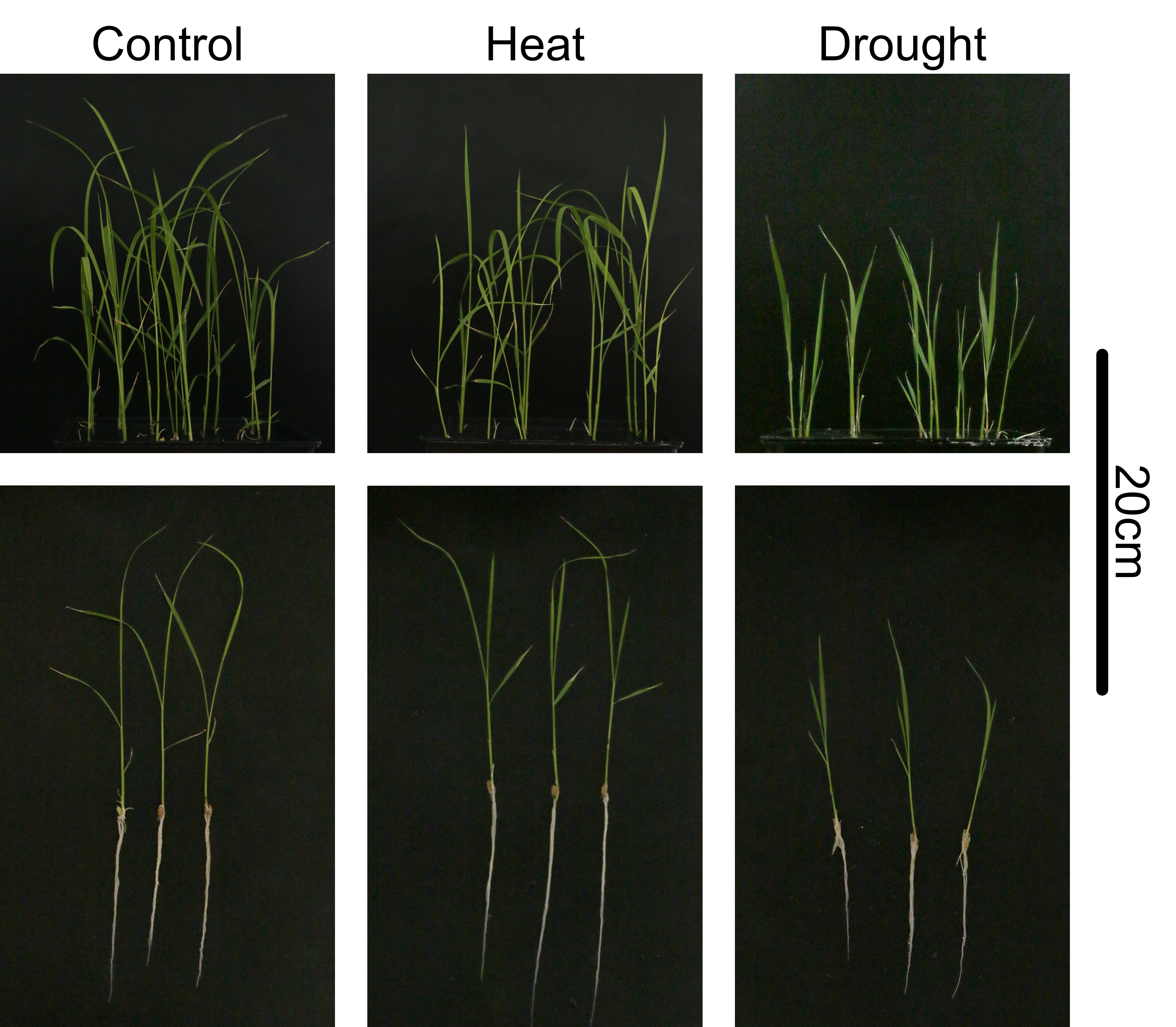

Supplement: Supplemental Information 9 — There was no significant change in rice phenotype after heat stress treatment. [file peerj-12-17255-s009.png]

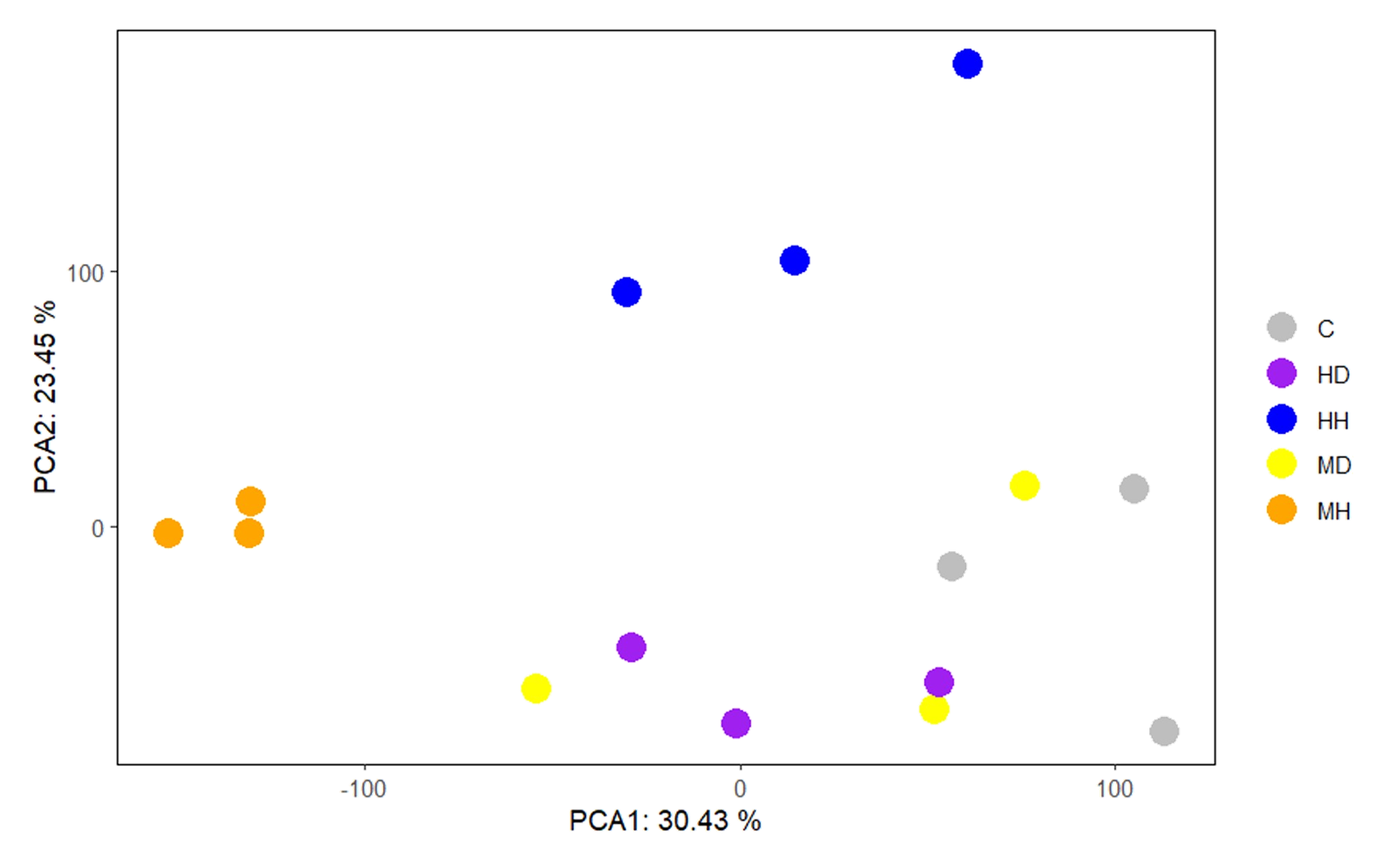

Supplement: Supplemental Information 10 — C, Control; MD, Moderate drought; HD, Severe drought; MH, Short-term time heat shock; HH, Long-term time heat shock. The Drought group is too close to the Control group. [file peerj-12-17255-s010.png]
